# Evaluation of *Babesia gibsoni* GPI-anchored Protein 47 (BgGPI47-WH) as a Potential Diagnostic Antigen by Enzyme-Linked Immunosorbent Assay

**DOI:** 10.3389/fvets.2019.00333

**Published:** 2019-10-02

**Authors:** Xueyan Zhan, Long Yu, Xiaomeng An, Qin Liu, Muxiao Li, Zheng Nie, Yangnan Zhao, Sen Wang, Yangsiqi Ao, Yu Tian, Lan He, Junlong Zhao

**Affiliations:** ^1^State Key Laboratory of Agricultural Microbiology, College of Veterinary Medicine, Huazhong Agricultural University, Wuhan, China; ^2^Key Laboratory of Preventive Veterinary Medicine in Hubei Province, Wuhan, China; ^3^Key Laboratory of Development of Veterinary Diagnostic Products, Ministry of Agriculture of the People's Republic of China, Wuhan, China

**Keywords:** *Babesia gibsoni*, GPI-anchored protein, ELISA, diagnosis, babesiosis

## Abstract

*Babesia gibsoni* is one of the important pathogens causing severe incurable canine babesiosis, suggesting the necessity to develop a sensitive, specific, and highly automated diagnostic method for clinical application. Surface proteins are ideal candidates for diagnostic targets because they are the primary targets for host immune responses during host-parasite interactions. Glycosylphosphatidylinositol (GPI)-anchored proteins are abundant on the surface of parasites and play an important role in parasite diagnosis. In this study, a GPI-anchored protein named BgGPI47-WH was obtained and mouse anti-rBgGPI47-WH polyclonal antibody was produced by immunizing mice with the purified protein and Freund's adjuvant. Western blot was used to identify the native form and immunogenicity of BgGPI47-WH. An ELISA method was established by using recombinant BgGPI47-WH protein to evaluate its potential as a diagnostic antigen and the established method exhibited high specificity. The antibody response was evaluated by using the *B. gibsoni*-infected sera collected from different experimental dogs and the established ELISA could recognize antibodies at day 6 until day 101 post infection, indicating the potential use of BgGPI47-WH for early stage diagnosis. The specificity of the established ELISA was further evaluated by using 147 clinical samples collected from animal hospitals and 17.0% (25/147) of the samples were tested positive, with an overall proportion agreement of 86.39% between the results from BgGPI47-WH and BgSA1. Our results indicated that BgGPI47-WH could be used as a reliable diagnostic antigen and this study has proposed a practical method for early diagnosis of *B. gibsoni*.

## Introduction

Canine babesiosis is caused by tick-transmitted *Babesia* species and geographically spread in dogs in China, with its distribution matching that of its vector tick. *Babesia gibsoni* (*B. gibsoni*) is one of the predominant *Babesia* species causing canine babesiosis in dogs. Since its first discovery in India in 1910, *B. gibsoni* has been reported in Asia, Australia, and even Europe (1, 2). Studies have shown that it can spread through ticks, blood transfusion, and placenta (2, 3). In 2017, He et al. reported the strain of *B. gibsoni* in Wuhan and provided the molecular biological evidence such as 18S rRNA and ITS gene sequence, confirming the epidemic of *Babesia* in Wuhan from the evolutionary point of view (4).

The diagnosis of *B. gibsoni* mainly depends on blood smear microscopy, but this method can be difficult for diagnosis of chronic infection with a low erythrocyte infection rate and is not suitable for epidemiological research of a large number of samples. Additionally, a dog, once infected with *B. gibsoni*, will become premonition immunity due to its vulnerability to relapse. This suggests the necessity to develop a simple and fast diagnostic method for clinical application. To this end, it is of great significance to obtain some specific proteins for clinical diagnosis of dogs and epidemiological investigation.

Parasite surface proteins are generally recognized as prominent candidates for development of diagnosis method, because they are the primary targets for host immune responses during host-parasite interactions (5). There are many proteins on the surface of *B. gibsoni* with an extracellular form. Some of them are attached to the parasite surface by glycosylphosphatidylinositol (GPI) anchor as exposed and neutralized antibodies in the host (6). Additionally, GPI-anchored proteins could be enzymatically removed from GPI anchors by phosphatidylinositol-specific phospholipase C (PI-PLC) and released into the extracellular environment to participate in host-parasite interactions (7). GPI-anchored proteins are relatively hydrophilic and most of them are dominant vaccine candidates.

Currently, a total of 19 GPI-anchored proteins have been screened in *B. microti*, and 17 of them were successfully expressed and identified as potential diagnostic markers. Additionally, each of them was proved to react with *B. microti*-infected serum. The microarray antibody assay confirmed that BmGPI12 has the highest sensitivity and specificity with the greatest potential as a new diagnostic marker and drug target (8). In *B. bovis*, an ELISA method was successfully established using MSA-2c with a satisfactory detection result (9). However, there are few reports on GPI-anchored proteins in *Babesia-*infected dogs. Only the Bc28 multigene family has been reported to play a crucial role in merozoite surface antigens in *B. canine* (10–12). This implies that GPI-anchored proteins may play a vital role in detection of *Babesia*.

To our best knowledge, there is no commercial diagnostic kit available for identifying cases of *Babesia* in China. The identification of GPI-anchored genes not only facilitates the treatment of *Babesia*, but also provides new targets for the development of sensitive, specific, and highly automated detection methods. It will be useful for the early detection of *Babesia*, and contribute to reducing the health burden of *B. gibsoni* in dogs.

The purpose of this study was to use bioinformatics methods to screen GPI-anchored proteins, followed by cloning and expressing the target protein and establishing an ELISA method to evaluate its possibility as a potential diagnostic antigen. This study will help us to surveille *B. gibsoni* infection in dogs, start treatment as early as possible, and control the epidemic of dog babesiosis.

## Methods

### Bioinformatics Analysis

The surface antigens of *B. gibsoni* were searched in the database websites (https://www.ncbi.nlm.nih.gov/; http://www.uniprot.org/; http://piroplasmadb.org/piro/) by bioinformatics. The results were blasted with *B. gibsoni* (Wuhan strain) genome to screen the potential surface proteins. GPI-anchor sequences were predicted by bigPI (http://mendel.imp.ac.at/gpi/plant_server.html), PredGPI (http://gpcr.biocomp.unibo.it/predgpi/), and GPI-SOM (http://gpi.unibe.ch/). As a result, **five** *B. gibsoni* (Wuhan strain) GPI-anchored protein genes were obtained, which were named as BgGPI50-WH, BgGPI47-WH, BgGPI45-WH, BgGPI32-WH, and BgGPI12-WH, respectively. All the five candidate antigens were found to have GPI anchor sites at the C-terminus, signal peptides, and multiple antigenic epitopes, suggesting their high potential as diagnostic markers.

### Experimental Animals and Strain

Three Beagle dogs were purchased from Anlu Laboratory Animal Center and numbered as A, B, and C, respectively. All dogs were confirmed to be *Babesia* free by PCR (13). *B. gibsoni* (Wuhan strain) was preserved in our laboratory. The clinical samples were collected from animal hospitals in Wuhan, China.

### gDNA and Total RNA Extraction

Genomic DNA was extracted from the blood samples by using the TIANamp Genomic DNA Kit (TIANGEN Biotech, Beijing, China) according to the manufacturer's instructions and stored in −20°C until further use.

Total RNA was extracted from 400 μL leukocyte-free *B. gibsoni*-infected dog blood by using TRIzol®RNA (Invitrogen, California, USA) following the manufacturer's instructions. The RNA was converted into cDNA by reverse-transcribed PCR (RT-PCR) using the Fast Quant® RT Kit (TIANGEN Biotech, Beijing, China), and stored at −80°C until further use.

### Cloning and Sequencing of BgGPI47-WH

The sequence of *B. gibsoni* P47 gene (BgGPI47-WH) was amplified using both gDNA and cDNA from *B. gibsoni* as a template with the specific primers (BgGPI47-WH-F/BgGPI47-WH-R) (Table 1). The thermal cycling parameters included the activation of TransStart KD plus DNA Polymerase at 94°C for 5 min, 35 cycles of (denaturation at 94°C for 30 s, annealing at 60°C for 30 s, extension at 68°C for 1 min 30 s), and a final extension at 68°C for 10 min. The ORF sequence of BgGPI47-WH was amplified from cDNA with specific primers (BgGPI47-WH-*BamH* I-F/BgGPI47-WH-*Xho* I-R) (Table 1) using the same PCR procedure as described above.

**Table 1:** Primers used for the amplification of the partial BgGPI47-WH genes.

**Primers**	**Primer sequences (5^**′**^-3^**′**^)**	**Restriction enzyme**
BgGPI47-WH-F	5′-ATGAAAGTCATTAATACTTTCCTTCTATTC-3′	
BgGPI47-WH-R	5′-TTAAAATACAGCGACAGCCACAG-3′	
BgGPI47-WH-*BamH* I-F	5′-CAGGATCCAATGGAGAAGATCAGGAAACAG-3′	*Bam*H I
BgGPI47-WH-*Xho* I-R	5′-CTCGAGTTAAAATACAGCGACAGCCACAG-3′	*Xho* I

*Underline represents the restriction enzyme sites*.

The lengths of the fragments were confirmed on 0.8% agarose gel (TSINGKE Biological Technology, Beijing, China), and then purified by Easy Pure® PCR Purification Kit (TransGEN, Beijing, China). The purified products were cloned into a pEASY-Blunt vector (TaKaRa Biotechnology, Beijing, China) and then ligated into a pET-28a expression vector. All the constructs were confirmed by DNA sequencing.

### Expression of Recombinant Proteins

After cloning BgGPI47-WH gene into the expression vector pET-28a with restriction enzyme *Bam*HI and *Xho*I, his-tagged recombinant proteins (rBgGPI47-WH) were expressed in Transetta (DE3) *E. coli* cells. The soluble proteins were purified by using ProteinPure Ni-NTA Resin (TransGEN Biotech, Beijing, China) according to the manufacturer's instructions.

### Antibody Generation

Five KunMing mice were purchased from the Laboratory Animal Center of Huazhong Agricultural University, and anti-rBgGPI47-WH serum was raised in them 7 days later. The mice were subcutaneously immunized with 100 μg of rBgGPI47-WH and Freund's complete adjuvant (Sigma, Shanghai, China) in the first immunization. The second immunization was performed with the same amount of rBgGPI47-WH protein emulsified with Freund's incomplete adjuvant (Sigma, Shanghai, China) after an interval of 14 days. The third and fourth immunization were carried out using the same procedure for the second immunization. The sera were collected and total immunoglobulin Gs (IgGs) were purified from the sera by using Protein G chromatography column (Beyotime Biotechnology, Shanghai, China) according to the manufacturer's instructions and then stored at −20°C until further use.

### Preparation of *B. gibsoni* Lysates and Western Blot Analysis

*Babesia gibsoni* lysates were prepared as previously reported [30]. Briefly, the normal/infected erythrocytes were lysed with red blood cell lysate buffer (Tris/EDTA/NaCl), and the precipitate was collected after several washes with PBS, followed by storage at −20°C for further use.

The recombinant rBgGPI47-WH samples were electrophoresed on 12% SDS-PAGE gels and then transferred to nitrocellulose membranes (Merck, New Jersey, USA) using the Semi-Dry blotting system. *B. gibsoni* positive serum was used as the primary antibody, and HRP-labeled goat anti-canine IgG (Southern Biotech, USA) was used as the secondary antibody.

In order to identify native BgGPI47-WH, *B. gibsoni*-infected and normal canine erythrocyte lysates were collected and reacted first with mice anti-rBgGPI47-WH IgG, then with HRP-labeled goat anti-mouse IgG, the secondary antibody. The antibody reactive bands were detected by an electrochemiluminescence (ECL) method. Preimmune mice serum was used as the negative control.

### Establishment of ELISA Method

An indirect ELISA was developed by using rBgGPI47-WH in 96-well plates as follows. (1) rBgGPI47-WH was diluted to an appropriate concentration with a coating buffer (25 mmol/L carbonate buffer solution, pH 9.6), followed by adding 100 μL diluted rBgGPI47-WH to 96-well plates and incubation at 4°C overnight. (2) The 96-well plates were washed three times with washing buffer (0.5% Tween-20 in PBS, pH 7.2), followed by the addition of 150 μL blocking buffer (1% BSA in washing buffer) into the plates and incubation at 37°C (3) After three washes with the washing buffer, 100 μL serum was added and incubated at 37°C. (4) After four washes with the same buffer, the secondary antibody HRP-labeled goat anti-canine was added into the wells, followed by incubation at 37°C and then four washes with the same buffer. (5) Then, the plates were supplemented with 100 μL TMB (Tetramethylbenzidine) in substrate buffer containing 0.022% H_2_O_2_. Finally, the reaction was terminated by adding 0.25% HF and the 96-well plates were read at 630 nm in an ELISA reader.

### Specificity Assay

Six different sera were collected for specificity assay, including positive serum of *Babesia canine* (*B. canine*), *Toxoplasma gondii* (*T. gondii*), Canine rabies virus (RABV), *Strongyloides stercoralis* (*S. stercoralis*), Dog hydatid, and Canine parvovirus (CPV). Meanwhile, the results were compared to those detected by ELISA based on BgSA1-WH.

### Antibody Response to rBgGPI47-WH in Experimentally Infected Dogs

The *B. gibsoni*-infected erythrocyte was withdrawn from the liquid nitrogen container and resuscitated in a water bath at 37°C. Each Beagle dog was intravenously infected with 5 mL *B. gibsoni*-infected blood and the parasitemia was detected every 3 days. Serum samples were collected every 3 or 7 days until day 101 post infection and stored at −20°C for further analysis. All Beagle dogs in this experiment were treated strictly according to standard animal welfare requirements.

### Diagnosis of Clinical Samples

A total of 147 serum samples were collected from domestic dogs in animal hospitals in Wuhan, China and detected by the established ELISA method. Meanwhile, the results were compared to those of another iELISA based on BgSA1 (unpublished data). All the results were repeated at least three times. The overall proportion agreement between the results of BgGPI47-WH ELISA and BgSA1-WH ELISA was determined by using an online tool, EpiTools epidemiological calculators (http://epitools.ausvet.com.au/content.php?page=Compare2Tests).

## Results

### BgGPI47-WH Sequence Analysis

**Figure 1:**
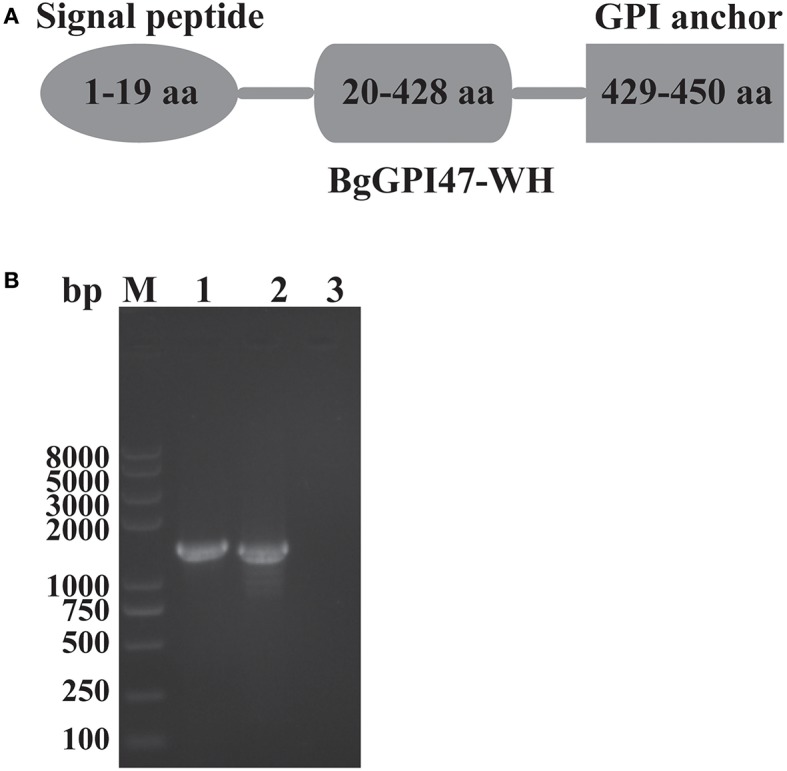
Sequence analysis of BgGPI47-WH. **(A)** Graphic depiction of BgGPI47-WH ORF. The sequence of BgGPI47-WH includes a signal peptide at the N-terminus and a typical GPI anchor at the C-terminus. **(B)** The PCR amplification of BgGPI47-WH genes from gDNA and cDNA of *B. gibsoni*. Lane M: Marker; lane 1: The BgGPI47-WH from gDNA; lane 2: The BgGPI47-WH from cDNA; lane 3: Negative control.

The sequences of BgGPI47-WH were amplified from both gDNA and cDNA by PCR using the specific primers shown in Table 1, and they were 1,390 and 1,353 bp, respectively (Figure 1B), with a 37 bp intron in BgGPI47-WH gene from 338 to 374 bp. The open reading frame sequence of BgGPI47-WH encodes 450 aa with a predicted size of 49 kDa. Sequence analysis showed that BgGPI47-WH has a signal peptide at the N-terminus from 1 to 19 aa and a typical GPI anchor at the C-terminus from 429 to 450 aa (Figure 1A). NCBI blast showed that the sequence of BgGPI47-WH gene was most similar (99.78%) to that of *B. gibsoni* strains in dogs in Taiwan.

### Expression of Recombinant Proteins

**Figure 2:**
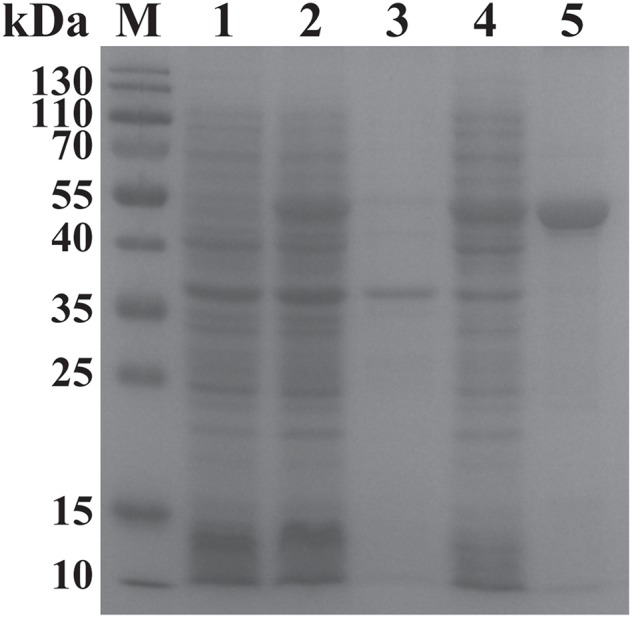
SDS-PAGE analysis of the recombinant BgGPI47-WH. Lane M: Protein marker; lane 1: Induced protein; lane 2: Non-induced protein; lane 3: Precipitated his-BgGPI47-WH in cell lysates; lane 4: Soluble his-BgGPI47-WH in cell lysates; lane 5: Purified recombinant BgGPI47-WH protein.

The ORF of BgGPI47-WH was obtained from *B. gibsoni* cDNA by PCR (Figure 1B) and was then ligated to pET-28a expression vector. The plasmid with correct sequencing was transferred into *E. coli* transetta (DE3) and induced for expression. The recombinant BgGPI47-WH (rBgGPI47-WH) containing a His-tag was purified by ProteinPure Ni-NTA Resin with a 54 kDa band (Figure 2). Meanwhile, the protein was collected and used to generate antibody.

### Identification of Native BgGPI47-WH and Antigenicity Analysis by Western Blot

**Figure 3:**
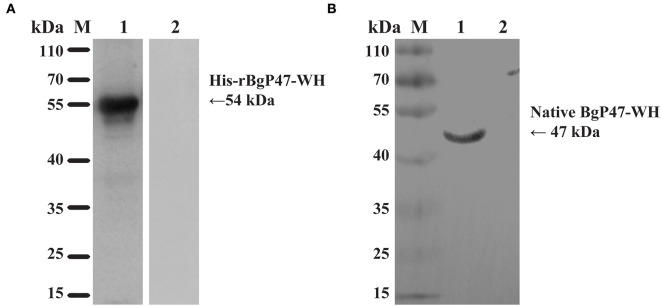
Western blot analysis of BgGPI47-WH. **(A)** Determination of antibody response of rBgGPI47-WH in *B. gibsoni*-infected canine serum. Lane M: Molecular weight marker; lane 1: rBgGPI47-WH reacted with *B. gibsoni*-infected canine serum; Lane 2: rBgGPI47-WH was probed with normal serum from non-infected canine serum. **(B)** Identification of native BgGPI47-WH in *B. gibsoni*. Lane M: Molecular weight marker; lane 1: *B. gibsoni*-infected canine RBC lysates were probed with mouse anti-BgGPI47-WH serum; lane 2: Non-infected canine RBC lysates were probed with mouse anti-BgGPI47-WH serum.

To identify the native BgGPI47-WH protein in *B. gibsoni*, anti-rBgGPI47-WH polyclonal sera were raised in Kunming mice. After purification using a Protein G chromatography column, the polyclonal sera were used to detect *B. gibsoni* lysates. Western blot results revealed one band of ~47 kDa in the lysate of *B. gibsoni*-infected RBCs (Figure 3B), but no signal was observed in the lysates of non-infected canine RBCs and preimmune serum (data not shown).

The specific antigenicity of BgGPI47-WH was tested by Western blot. After the reaction of the BgGPI47-WH protein with the positive serum from *B. gibsoni*-infected canine, using the non-infected canine serum as a control, the specific band of BgGPI47-WH was detected in the *B. gibsoni*-infected canine serum, but not in the non-infected canine serum (Figure 3A).

### Determination of Optimal Reaction Conditions for the ELISA Method

Following the above steps, we established an ELISA method and optimized the conditions. The optimal concentration of protein or antibodies for ELISA was determined by preliminary titration experiments, which was 0.25 μg/mL of BgGPI47-WH specific GPI-anchored protein in coating buffer. And the plates were blocked with 150 μL/well blocking buffer for 30 min at 37°C. The canine serum diluted at 1: 200 was used as the primary antibody (100 μL/well) and incubated for 1 h at 37°C. Then HPR-labeled goat anti-canine diluted at 1: 8000 was used as the secondary antibody (100 μL/well) and incubated for 1 h at 37°C. Each well was supplemented with 100 μL/well TMB containing 0.022% H_2_O_2_ in substrate buffer and incubated for 10 min at room temperature. After terminating, the 96-well plates were read at 630 nm in an ELISA reader.

### Determination of Specificity of BgGPI47-WH-ELISA

**Figure 4:**
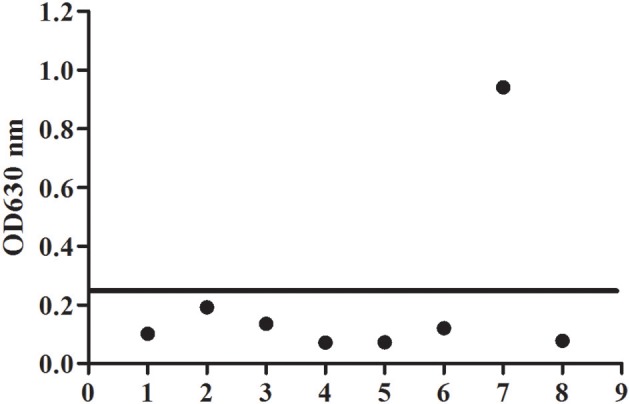
Determination of specificity of BgGPI47-WH-ELISA. Lane 1: *Strongyloides stercoralis* -infected dog sera; lane 2: Dog hydatid-infected dog sera; lane 3: Canine parvovirus-infected dog sera; lane 4: Canine rabies virus-infected dog sera; lane 5: *Toxoplasma gondii*-infected dog sera; lane 6: *Babesia canine*-infected dog sera; lane 7: Positive control; lane 8: Negative control.

To evaluate the potential of the rBgGPI47-WH expressed in *E. coli* as a diagnostic antigen for *B. gibsoni* infection, the purified rBgGPI47-WH was used to establish an ELISA method. The specificity of the ELISA method was evaluated by using the canine samples experimentally infected with *B. canine, T. gondii, S. stercoralis*, RABV, Dog hydatid, and CPV. All samples showed negative except *B. gibsoni* positive serum (Figure 4). The results indicated that the established ELISA has no cross reactivity with the positive dog serum of *B. canine, T. gondii, S. stercoralis*, RABV, Dog hydatid, and CPV. Meanwhile, BgSA1-WH ELISA showed no cross reactivity with the positive dog serum of *S. stercoralis* and RABV (unpublished data). Obviously, BgGPI47-WH ELISA is more specific than BgSA1-WH ELISA.

### Antibody Response to rBgGPI47-WH in Experimental Dogs Infected With *B. gibsoni*

**Figure 5:**
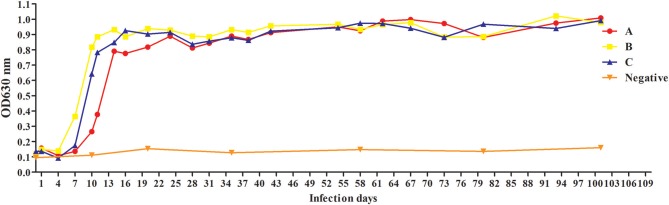
Antibody response to rBgGPI47-WH in dogs experimentally infected with *B. gibsoni*. The dog B experimentally infected with *B. gibsoni* exhibited a significant antibody response to the BgGPI47-WH specific antigen on the sixth day post infection. The dogs A and C showed a significant antibody response to the BgGPI47-WH specific antigen on the eighth day post infection. The antibody response of all the three experimental dogs remained at high levels until 101 days after infection with *B. gibsoni*.

To evaluate antibody response to rBgGPI47-WH, we collected serum samples from experimental dogs infected with *B. gibsoni* until day 101 post infection. The antibody response was detected by the established ELISA method based on rBgGPI47-WH. The results showed that the experimental dog B infected with *B. gibsoni* exhibited a significant antibody response to the BgGPI47-WH specific antigen on the sixth day post infection. Meanwhile, experimental dogs A and C showed a significant antibody response to the BgGPI47-WH specific antigen on the eighth day post infection. The antibody continued to rise from day 6 and 8 to day 11 and 20 until reaching a relatively stable level, respectively. The antibody response of all the three experimental dogs remained at high levels until day 101 after infection with *B. gibsoni*, and even at a premonition stage of infection, while the three dogs showed a significantly low level of parasitemia (Figure 5).

### Diagnosis of Clinical Samples by the ELISA Based on BgGPI47-WH and BgSA1

A total of 147 samples collected from animal hospitals in Wuhan, China were detected by ELISA method based on both BgGPI47-WH and BgSA1. Twenty-five of the 147 samples were tested positive by BgGPI47-WH ELISA (17.0%). The overall proportion agreement between the two ELISA methods was calculated to be 86.39% by EpiTools.

## Discussion

*B. gibsoni* is an important causative agent of canine babesiosis in the world, and early diagnosis of the disease plays an increasingly important role in the pet industry. In this study, we discovered a GPI-anchored protein termed as BgGPI47-WH in *B. gibsoni* by the bioinformatics method. The sequence of BgGPI47-WH was amplified from gDNA and cDNA of *B. gibsoni* using specific primers. After sequencing, it was shown to contain a 37 bp intron in the BgGPI47-WH from the gDNA of *B. gibsoni*. Bioinformatics analysis showed that the BgGPI47-WH sequence includes a signal peptide at the N-terminus and a typical GPI anchor at the C-terminus. The amino acid sequence shared 99.78% homology with that of BgP47 reported by Aboge et al. (14), and 41.05% homology with that of BgP50 reported by Liu et al. (15). BgGPI47-WH was predicted by DNAStar Protean software to have a high antigen index and a high surface probability. All these bioinformatics analysis results indicated that BgGPI47-WH could be a potential serodiagnostic antigen. Then, BgGPI47-WH was ligated into the plasmid pET-28a and induced for expression.

Surface proteins are considered as good potential vaccine candidates and diagnosis antigens, and have been confirmed to play important roles in the invasion of host cells in Apicomplexan protozoan (16–18). In *Plasmodium falciparum*, the GPI-anchored circumsporozoite protein (CSP) and merozoite surface proteins (MSP-1 and-2) of sporozoites and schizonts have been shown to protect rodents from invasion in varying degrees (19). In *B. bovis*, variable merozoite surface antigens (VMSAs) are important vaccine candidates to control *B. bovis*, and their antibodies could neutralize the invasion of red blood cells *in vitro* (20). GPI-anchored proteins have also been identified in *Babesia-*infected dogs, such as the Bc28 family (10–12). In *Toxoplasma gondii*, the SAG family has also been identified as an ideal diagnostic antigen and vaccine candidate (21).

P47 has been reported as a surface protein (14, 22), and the sequence of BgGPI47-WH is only one amino acid different from the reported sequence, probably due to the large genetic difference between geographical isolates. The BgGPI47-WH protein was found to be expressed in the supernatant, while the recombinant protein BgP47 of the Japanese strain *B. gibsoni* was reported as an insoluble protein (22). All these reports indicated that the BgGPI47-WH of Wuhan strain *B. gibsoni* has a higher possibility of becoming a diagnostic marker than the BgP47 of the Japanese strain *B. gibsoni*. Western blot analysis showed that the BgGPI47-WH protein could differentiate *B. gibsoni*-infected positive canine serum from non-infected negative canine serum (Figure 3A), indicating the specific antigenicity of BgGPI47-WH. Immunoblotting analysis of the native BgGPI47-WH revealed a 47 kDa band, corresponding to the expected size. All the results showed that BgGPI47-WH could be a potential diagnosis antigen.

Recently, *B. gibsoni* has attracted renewed interest due to its widespread in the world, and it is necessary to develop a sensitive and specific detection method for screening potentially infected animals to prevent the spread of the disease. The ELISA method is easier to operate and has a higher specificity for a large number of samples. BgTRAP has been reported as a good candidate antigen for serological diagnosis by indirect ELISA (23), but it is difficult to express and has a low sensitivity, suggesting the urgency for developing more prospective antigens (23, 24). Researchers have identified many surface proteins in a cDNA library and confirmed most of them as important candidates for developing a diagnostic method to control *B. gibsoni* infection in dogs (25). In the present study, we established a feasible ELISA method with rBgGPI47-WH, which was able to differentiate clinically canine samples infected with *B. canine, T. gondii, S. stercoralis*, RABV, Dog hydatid, and CPV, with no cross reaction observed between rBgGPI47-WH and the positive serum of the above six antigens. Meanwhile, the ELISA with rBgGPI47-WH could specifically recognize the antibody against BgGPI47-WH from the sera collected from dogs experimentally or naturally infected with *B. gibsoni*. In the serum samples derived from dogs experimentally infected with *B. gibsoni*, the antibody response to BgGPI47-WH became positive for dog B on day 6 post infection, while the other two dogs became positive on day 8 post infection. The results are consistent with the report by Fukumoto et al. (26), confirming that BgGPI47-WH is a good candidate for early stage diagnosis of dog *B. gibsoni* infection.

## Conclusions

In this study, a GPI-anchored protein was obtained from *B. gibsoni* and named as BgGPI47-WH. Western blot analysis showed that BgGPI47-WH has a high immunogenicity and an ELISA method was developed to evaluate its potential as a diagnostic antigen. The results showed that the established method has a high specificity and could recognize the antibody against BgGPI47-WH from day 6 to day 101 post infection. All results showed that BgGPI47-WH could be a potential diagnostic antigen for early stage diagnosis of *B. gibsoni* infection and contribute to the prevention of its prevalence.

## Data Availability Statement

The nucleotide sequence was annotated and submitted to the NCBI GenBank under the accession number MK943815.

## Ethics Statement

The experimental animals were raised in an enriched environment. All experimental animals are kept in a rich environment based on the regulations governing the management of laboratory animals in the People's Republic of China. All experiments were carried out in accordance with the guidelines set by Huazhong Agricultural University and approved by the Animal Welfare and Research Ethics Statement of Huazhong Agricultural University (license number: HZAUDO-2017-005).

## Author Contributions

XZ, LH, and JZ designed the study and wrote the draft of the manuscript. LY, XA, QL, ML, ZN, YZ, SW, YA, and YT performed the experiments and analyzed the results. All authors have read and approved the final manuscript.

### Conflict of Interest

The authors declare that the research was conducted in the absence of any commercial or financial relationships that could be construed as a potential conflict of interest.

## References

[1] Groves MG Yap LF. Babesia gibsoni (Patton, 1910) from a dog in Kuala Lumpur. Med J Malaya. (1968) 22:229. 4234362

[2] Kjemtrup AM Kocan AA Whitworth L Meinkoth J Birkenheuer AJ Cummings J. There are at least three genetically distinct small piroplasms from dogs? Int J Parasitol. (2000) 30:1501–5. 10.1016/S0020-7519(00)00120-X 11428342

[3] Birkenheuer AJ Correa MT Levy MG Breitschwerdt EB. Geographic distribution of babesiosis among dogs in the United States and association with dog bites: 150 cases (2000–2003). J Am Vet Med Assoc. (2005) 227:942–7. 10.2460/javma.2005.227.942 16190594

[4] He L Miao X Hu J Huang Y He P He J. First molecular detection of Babesia gibsoni in dogs from Wuhan, China. Front Microbiol. (2017) 8:1577. 10.3389/fmicb.2017.01577 28871243 PMC5566568

[5] T.Schetters PM Montenegro-James S. Vaccines against babesiosis using soluble parasite antigens. Parasitol Today. (1995) 11:456–62. 10.1016/0169-4758(95)80059-X 15275383

[6] Nathaly Wieser S Schnittger L Florin-Christensen M Delbecq S Schetters T. Vaccination against babesiosis using recombinant GPI-anchored proteins. Int J Parasitol. (2019) 49:175–81. 10.1016/j.ijpara.2018.12.002 30684517

[7] Rodriguez AE Florin-Christensen M Flores DA Echaide I Suarez CE Schnittger L. The glycosylphosphatidylinositol-anchored protein repertoire of *Babesia bovis* and its significance for erythrocyte invasion. Ticks Tick Borne Dis. (2014) 5:343–8. 10.1016/j.ttbdis.2013.12.011 24642346

[8] Cornillot E Dassouli A Pachikara N Lawres L Renard I Francois C. A targeted immunomic approach identifies diagnostic antigens in the human pathogen Babesia microti. Transfusion. (2016) 56:2085–99. 10.1111/trf.13640 27184823 PMC5644385

[9] Bono MF Mangold AJ Baravalle ME Valentini BS Thompson CS Wilkowsky SE. Efficiency of a recombinant MSA-2c-based ELISA to establish the persistence of antibodies in cattle vaccinated with Babesia bovis. Vet Parasitol. (2008) 157:203–10. 10.1016/j.vetpar.2008.07.025 18783887

[10] Carcy B Randazzo S Depoix D Adaszek L Cardoso L Baneth G. Classification of *Babesia canis* strains in Europe based on polymorphism of the Bc28.1-gene from the *Babesia canis* Bc28 multigene family. Vet Parasitol. (2015) 211:111–23. 10.1016/j.vetpar.2015.05.028 26092188

[11] Eichenberger RM Ramakrishnan C Russo G Deplazes P Hehl AB. Genome-wide analysis of gene expression and protein secretion of *Babesia canis* during virulent infection identifies potential pathogenicity factors. Sci Rep. (2017) 7:3357. 10.1038/s41598-017-03445-x 28611446 PMC5469757

[12] Yang YS Murciano B Moubri K Cibrelus P Schetters T Gorenflot A. Structural and functional characterization of Bc28.1, major erythrocyte-binding protein from *Babesia canis* merozoite surface. J Biol Chem. (2012) 287:9495–508. 10.1074/jbc.M111.260745 22294693 PMC3308747

[13] He L Feng HH Zhang WJ Zhang QL Fang R Wang LX. Occurrence of Theileria and Babesia species in water buffalo (Bubalus babalis, Linnaeus, 1758) in the Hubei province, South China. Vet Parasitol. (2012) 186:490–6. 10.1016/j.vetpar.2011.11.021 22154255

[14] Aboge GO Batbaatar V Goo YK Yamagishi J Nishikawa Y Sunaga F Molecular characterization and expression of a 47-kDa merozoite surface protein of *Babesia gibsoni* for serodiagnosis by enzyme-linked immunosorbent assay. J Protozool Res. (2010) 20:59–69.

[15] Liu M Cao S Zhou M Wang G Jirapattharasate C Moumouni PFA. Genetic variations of four immunodominant antigens of Babesia gibsoni isolated from dogs in southwest Japan. Ticks Tick Borne Dis. (2016) 7:298–305. 10.1016/j.ttbdis.2015.11.005 26615873

[16] Hafalla JC Silvie O Matuschewski K. Cell biology and immunology of malaria. Immunol Rev. (2011) 240:297–316. 10.1111/j.1600-065X.2010.00988.x 21349101

[17] Goodswen SJ Kennedy PJ Ellis JT. A guide to *in silico* vaccine discovery for eukaryotic pathogens. Brief Bioinformatics. (2013) 14:753–74. 10.1093/bib/bbs066 23097412

[18] Man S Fu Y Guan Y Feng M Qiao K Li X. Evaluation of a major surface antigen of *Babesia microti* merozoites as a vaccine candidate against Babesia infection. Front Microbiol. (2017) 8:2545. 10.3389/fmicb.2017.02545 29312230 PMC5742146

[19] Plassmeyer ML Reiter K Shimp RL Jr Kotova S Smith PD Hurt DE. Structure of the *Plasmodium falciparum* circumsporozoite protein, a leading malaria vaccine candidate. J Biol Chem. (2009) 284:26951–63. 10.1074/jbc.M109.013706 19633296 PMC2785382

[20] Wilkowsky SE Farber M Echaide I Torioni de Echaide S Zamorano PI Dominguez M. Babesia bovis merozoite surface protein-2c (MSA-2c) contains highly immunogenic, conserved B-cell epitopes that elicit neutralization-sensitive antibodies in cattle. Mol Biochem Parasitol. (2003) 127:133–41. 10.1016/S0166-6851(02)00329-8 12672522

[21] Caetano BC Bruna ROB Mendes EA Penido ML Gazzinelli RT. Vaccination with replication-deficient recombinant adenoviruses encoding the main surface antigens of toxoplasma gondii induces immune response and protection against infection in mice. Human Gene Ther. (2006) 17:415. 10.1089/hum.2006.17.415 16610929

[22] Goo YK Aboge GO Terkawi MA Jia H Yamagishi J Sunaga F. Four promising antigens, BgP32, BgP45, BgP47, and BgP50, for serodiagnosis of *Babesia gibsoni* infection were classified as *B. gibsoni* merozoite surface protein family. Parasitol Int. (2012) 61:364–8. 10.1016/j.parint.2011.11.007 22172478

[23] Goo YK Jia H Aboge GO Terkawi MA Kuriki K Nakamura C. *Babesia gibsoni*: serodiagnosis of infection in dogs by an enzyme-linked immunosorbent assay with recombinant BgTRAP. Exp Parasitol. (2008) 118:555–60. 10.1016/j.exppara.2007.11.010 18155197

[24] Narantsatsral S Youn-Kyoung G Battsetseg B Myagmarsuren P Mohamad Alaa T Takehisa S. Expression of truncated *Babesia gibsoni* thrombospondin-related adhesive proteins in *Escherichia coli* and evaluation of their diagnostic potential by enzyme-linked immunosorbent assay. Exp Parasitol. (2011) 129:196–202. 10.1016/j.exppara.2011.07.011 21802417

[25] Goo YK Xuan X. New molecules in babesia gibsoni and their application for diagnosis, vaccine development, and drug discovery. Korean J Parasitol. (2014) 52:345–53. 10.3347/kjp.2014.52.4.345 25246713 PMC4170030

[26] Fukumoto S Xuan X Kadota K Igarashi I Sugimoto C Fujisaki K. High-level expression of truncated surface antigen P50 of Babesia gibsoni in insect cells by baculovirus and evaluation of its immunogenicity and antigenicity. Clin Diagn Lab Immunol. (2003) 10:596–601. 10.1128/CDLI.10.4.596-601.2003 12853391 PMC164249

